# Influence of experimental parameters on the laser heating of an optical trap

**DOI:** 10.1038/s41598-017-15904-6

**Published:** 2017-11-22

**Authors:** Frederic Català, Ferran Marsà, Mario Montes-Usategui, Arnau Farré, Estela Martín-Badosa

**Affiliations:** 1 Optical Trapping Lab – Grup de Biofotònica, Departament de Física Aplicada, Universitat de Barcelona, Martí i Franquès 1, Barcelona, 08028 Spain; 2 Institut de Nanociència i Nanotecnologia (IN2UB), Martí i Franquès 1, Barcelona, 08028 Spain; 3 Impetux Optics S. L., Trias i Giró 15 1-5, Barcelona, 08034 Spain

## Abstract

In optical tweezers, heating of the sample due to absorption of the laser light is a major concern as temperature plays an important role at microscopic scale. A popular rule of thumb is to consider that, at the typical wavelength of 1064 nm, the focused laser induces a heating rate of B = 1 °C/100 mW. We analysed this effect under different routine experimental conditions and found a remarkable variability in the temperature increase. Importantly, we determined that temperature can easily rise by as much as 4 °C at a relatively low power of 100 mW, for dielectric, non-absorbing particles with certain sets of specific, but common, parameters. Heating was determined from measurements of light momentum changes under drag forces at different powers, which proved to provide precise and robust results in watery buffers. We contrasted the experiments with computer simulations and obtained good agreement. These results suggest that this remarkable heating could be responsible for changes in the sample under study and could lead to serious damage of live specimens. It is therefore advisable to determine the temperature increase in each specific experiment and avoid the use of a universal rule that could inadvertently lead to critical changes in the sample.

## Introduction

Optical tweezers have been proven to be a powerful microtool for biological studies since their inception, pioneered by Arthur Ashkin^1^. This non-invasive technique exhibits some advantageous features including non-contact forces in the range 0.1–100 pN and compatibility with liquid medium environments which make it highly suitable for application in biological studies. However, even at the innocuous laser wavelength of 1064 nm used in our experiments, light absorption in water is not negligible; so localized heating at the focus of the optical trap and heat transfer to the immediate surroundings could produce small but significant thermal effects.

Different methods have been used to determine temperature increases due to the use of optical tweezers (see Supplementary Figure 6 and Supplementary Table 2). By means of the fluorescence emission shifts of a temperature-sensitive Laurdan dye probe, Liu *et al*.^2,3^ measured temperature increases of 1 °C/100 mW, 1.15 °C/100 mW and 1.45 °C/100 mW for trapped live human sperm cells, hamster ovary cells and liposomes, respectively. Haro-González *et al*.^4^ found an increase of 9.9 °C/100 mW for a 980-nm laser trap, using quantum dot luminescence thermometry. Ebert *et al*.^5^ developed a fluorescence ratio technique using the temperature sensitive dye Rhodamine B and the temperature-independent reference dye Rhodamine 110, and obtained a heating of 1.3 °C/100 mW. The same method was used by Wetzel *et al*., who determined an increase of 2.3 °C/100 mW^6^. Kuo^7^ used an adaptation of the wax-melting method to estimate temperature increase, which was found to be 1.7 °C/100 mW. The changes induced by temperature in the refractive index of water were monitored by Celliers *et al*.^8^, who measured a 4 °C temperature increase in a 55-mW, 985-nm laser trap (7.3 °C/100 mW).

Following a completely different approach, Peterman *et al*.^9^ introduced a technique based on the analysis of the thermal motion of a trapped bead and measured a temperature increase of 3 °C-4 °C/100 mW for different sizes of polystyrene beads diluted in glycerol and around 0.8 °C/100 mW for silica beads diluted in water. With the same method, Abbondanzieri *et al*.^10^, using a system based on a dual-beam optical trap, measured a heating of 0.4 °C every 100 mW of laser power at the back of the objective. Similarly, Jun *et al*.^11^ compared the active and passive calibration of an optical trap and inferred heating rates of 2.4 °C/100 mW and 1.2 °C/100 mW for 0.49-μm polystyrene and 0.64-μm silica beads, respectively, in a 980-nm laser trap. Finally, for a 975-nm low-NA laser trap, Mao *et al*.^12^ obtained 5.6 °C/100 mW by measuring the temperature-dependent viscosity of water in a Stokes drag experiment from direct force measurements based on light momentum.

Despite this variation in reported results, temperature increase at 1064 nm is often assumed, as a rule of thumb, to be approximately 1 °C/100 mW^13^. This small heating is then frequently used to argue for the relative innocuousness of laser traps, especially when used with live samples, such as cells. However, as discussed in ref.^9^, laser heating is directly dependent on the intensity distribution of the trapping laser and is therefore sensitive to changes in experimental conditions which could give rise to particularly unfavourable cases with considerably larger temperature increases. For example, Peterman *et al*.^9^ showed the increase in heating with the axial position of the trap in glycerol. Unfortunately, to the best of our knowledge, that is the only work in which the variability of the heating rate, *B*, is analysed with respect to some parameters.

To fill this gap, we assessed the change in *B* under different experimental conditions. Heating was measured while changing the numerical aperture of the objective, the suspension liquid, the material and size of the trapped particle, and the position of the trap. We found a remarkable dependence on some of the parameters and, more importantly, temperature increases as large as 4.0 °C/100 mW in water. The experimental scheme was based on the analysis of the variation in measured drag forces on trapped particles under different laser powers, similar to that adopted in refs^9,12^. The force was determined through measurement of the change in light momentum^14–16^. This method is independent of the specific properties of the sample; and particularly, it does not depend on the laser power or the chamber temperature. This allowed us to directly infer variations in the measured force as changes in the medium viscosity caused by the temperature rise. Our experiments were complemented with computer simulations of the heating produced by an optical trap. We used the model proposed by Peterman *et al*.^9^, which provided an accurate description of the experimental data.

## Results

### Measurement of temperature increase at the focus of the optical trap

**Figure 1:**
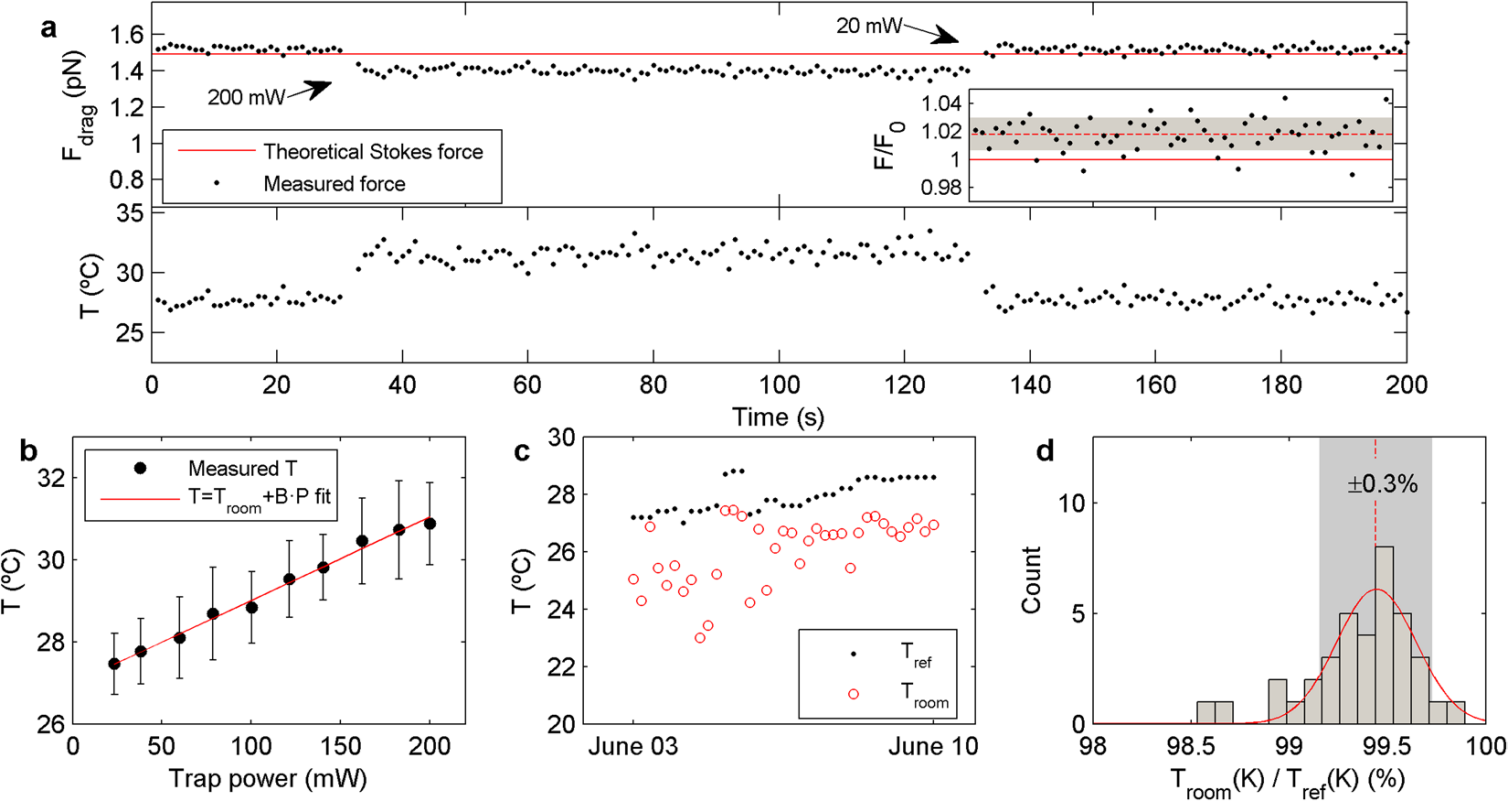
Determination of temperature through measurements of light momentum. (**a**) Top: Drag force measured on a 1.16-μm bead for 200 seconds. Each dot is the mean drag force obtained from the square force signal over one cycle, which is produced by the piezoelectric stage continuously applying a triangular oscillation. Power is switched from 20 mW to 200 mW at second 30 and back to 20 mW after 100 seconds. Bottom: Temperature obtained from the force measurement depicted in the top panel, through Eqs 1 and 2. (**b**) Both the heating rate, *B*, and room temperature, *T*_room_, were determined from the linear fit to measurements of temperature at 10 different laser powers between 20 and 200 mW. **(c)** Temperature values obtained from all the experiments on 1.16-μm beads in water (red circles) compared with independent measurements with a precision thermometer (black dots). **(d)** The ratio *T*_room_/*T*_ref_ showed a standard deviation similar to that expected from the ±2–3% of the diameter of the beads used.

In this work, determination of local temperature in an optical trap is based on the measurement of the viscosity of the solvent, which is accessible in a single step from Stokes-drag force measurements. As described in Methods and in the Supplementary Information, drag forces are assessed from light-momentum measurements, such that: F_drag_ = −*α*_detector_·S_x_, where S_x_ is the position sensitive detector (PSD) signal. The light-momentum calibration parameter, *α*_detector_, has been demonstrated to be independent of the geometry of the trapped object and of the structure of the trapping beam^14–17^, and importantly, it is not dependent on the laser power or sample temperature. In this way, changes in the measured drag force when incrementing the trap power, and therefore increasing the sample temperature, are directly caused by the variation in the medium viscosity, given as follows:

1 $$\eta (T)=\frac{{\alpha }_{\det ector}{S}_{x}}{6\pi Rvb}$$

where *R* is the radius of the trapped microsphere, *v* is the flow velocity and *b* is the Faxén correction due to a sphere-to-surface interaction^18^. With this intention, we used a piezoelectric stage to produce constant drag forces. In Fig. 1a (top), we show the sudden drop in the force reading when the trap power is switched from 20 mW to 200 mW. As all the other variables are fixed, this is indicative of a change in the water viscosity that arises from the temperature increase induced by laser heating. When the intensity is decreased back to its original value, the original force is reversibly recovered. Laser heating at and around the focus of an optical trap is due to absorption of the NIR laser light, primarily by the solvent^2,8,9,12^, as we discuss below. The dynamic viscosity thus becomes a natural vehicle to connect force readings and sample temperature. Variations in force readings can be translated into changes in the viscosity of the medium, which we can directly convert into changes of sample temperature (see Fig. 1a, bottom). For water, the relation between viscosity and temperature is given by^9^:

2 $$\mathrm{log}({\eta }_{{\rm{water}}}(T))=\frac{1.3272\cdot (293.15-T)-0.001053\cdot {(T-293.15)}^{2}}{T-168.15}-2.999$$

In agreement with previous results^2^, in all our experiments we found a linear relation between the trap power, *P*, and the temperature, *T*, in the range 20 °C–30 °C: *T* = *T*_room_ + *BP*, from which we determined the heating rate, *B* (°C/100 mW), and the room temperature, *T*_room_ (see Fig. 1b). In all the experiments, we swept the trap power from 20 mW to 200 mW (at every 20 mW) by properly setting the output laser power. As described in Methods, trap power was monitored from the *S*_SUM_ signal of the PSD.

We compared *T*_room_ with an independent measurement obtained with a precision thermometer (±0.1 °C), *T*_ref_, and observed clear correspondence between the measurements (Fig. 1c), with an average deviation of −1.5 °C (−0.5%) due to a slight discrepancy between the measured and the theoretical drag force (+2%). When normalized by *T*_ref_, the room temperature measurements exhibited a ±0.3% standard deviation (Fig. 1d), which mainly comes from the ±2–3% standard deviation of the diameter of the monodisperse polystyrene microspheres we used (see Supplementary Table 1).

### Experiments: dependence on experimental conditions

**Figure 2:**
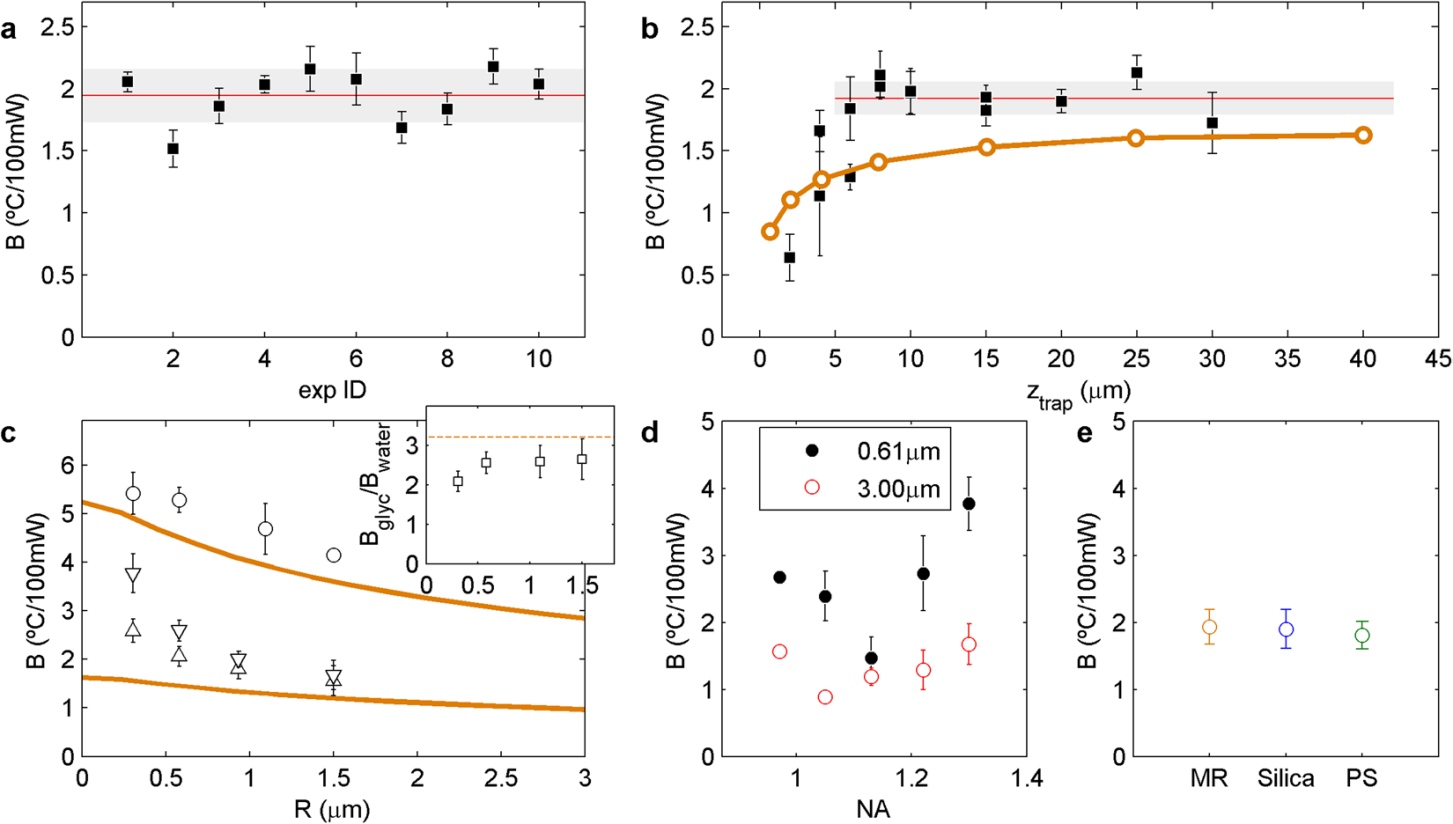
Variability of *B* under different experimental conditions. **(a)** Systematic measurements of the heating rate with the same sample showed a standard deviation of 11%, confirming the error estimation of 10–15% in our measurements. **(b)** Variation of *B* for different axial positions of the trap with a 1.16-μm polystyrene microbead (squares). The mean and standard deviation of the measured *B* values beyond *z*_trap_ *= *10 μm, equal to the independent measurement shown in **a**, are indicated by the red line and the grey area, respectively. Results from the simulations described in the next section are superimposed (orange trace). **(c)** Heating for particles of different radius, *R*, and the same material (polystyrene). Upward (downward) triangles correspond to particles trapped with the water- (oil-) immersion NA = 1.2 (NA = 1.3) objective in water, while circles correspond to the particles trapped with the NA = 1.2 objective in glycerol. The solid lines are radial temperature distributions obtained from simulations in water (*α*/*K = *23.7 °C/W) and glycerol (*α*/*K = *76.4 °C/W) respectively, both for the NA = 1.2 objective. The inset shows the experimental ratio *B*_glycerol_/*B*_water_ (squares), which is similar to the nominal ratio: 3.2 (dashed line). **(d)** Heating for 0.61-μm and 3.00-μm polystyrene microbeads in water, trapped with the oil-immersion objective with different values of NA_eff_. **(e)** *B* was found to be equal for three 2-μm particles of different materials (MR: melamine resin, PS: polystyrene).

Measurements of B, carried out as described in Methods and shown in Fig. 1b, had a reproducibility of approximately ±10% (compatible with that estimated from the linear fit), as shown in the experiment in Fig. 2a, which was repeated 10 times. Here, a 1.16-μm polystyrene microbead trapped at z_trap_ = 10 μm from the lower coverslip of the microchamber experienced a heating rate of 1.9 °C ± 0.2 °C/100 mW (±11%); similar to previous results in the literature (see Supplementary Figure 6 and Supplementary Table 2).

The first parameter analysed that affects trap heating was the axial position of the trap (Fig. 2b): a response previously measured and modelled in glycerol^9^. As discussed in the next section, the higher heat conductivity of the coverslip means that it acts as a heat sink, cooling the trap more as it is placed closer to the interface. Similarly to previous findings^9^, heating was observed to increase rapidly in the first 10 μm above the lower glass surface, though in our case it saturated at approximately 1.9 °C/100 mW (the same value obtained in Fig. 2a) in the range 10–30 μm. The temperature increase is chiefly concentrated in a region ~10–15 μm around the trap position, *z*_trap_, so that heat dissipation by the coverslip is actually sufficient to cool down the trap only below *z*_trap_ = 10–15 μm. For this experiment, the water-immersion, NA = 1.2 objective was used, which avoids spherical aberration and permits efficient trapping over the whole microchamber axial range.

We then analysed the effect of particle size. Despite it having been suggested that this parameter has only a small influence on the final result, we observed a difference of over twofold in *B* for particles with diameters from 0.61 μm to 3.00 μm (see Fig. 2c): much greater than the error associated with the determination of *B* (10–15%, see Fig. 2a). The larger the particle, the lower the heating was. As discussed in the next section, this result can be directly connected to the radial temperature profile caused by the optical trap. This was reproduced by both the water-immersion NA = 1.2 and the oil-immersion NA = 1.3 objectives, though greater heating was observed with the latter, especially for smaller beads.

In addition, we studied the effect of reducing the effective numerical aperture, NA_eff_, of the trapping beam. A diaphragm was placed at an optical equivalent to the entrance pupil of the NA = 1.3 objective (of focal length *f′*_obj_) to modify the diameter of the beam, *D*_beam_, such that: NA_eff_ = *D*_beam_/2 *f′*_obj_. The measurements were critically dependent on NA_eff_, exhibiting variations as large as ±2 °C/100 mW and ±1 °C/100 mW for 0.61-μm and 3.00-μm polystyrene beads, respectively (see Fig. 2d). For the case of 0.61-μm beads, our experimental results did not exhibit a monotonic trend; whereas they revealed an ascending pattern for 3.00-μm beads. The output laser power was set to produce a similar range for the trap power (20 mW to 200 mW) when reducing NA_eff_ (see Methods).

As reported by Peterman *et al*., *B* also depends on the suspension medium. The variation in temperature found when we changed water for glycerol was similar to the quotient between the ratio of light absorption, *α* (m^−1^), to thermal conductivity, *K*, between the two liquids, (*α*/*K*)_glycerol_/(*α*/*K*)_water_ = 3.2 (see Fig. 2c, inset). This result suggests that, as assumed by different models^2,8,9,12^, the temperature increase depends linearly on *α*/*K*.

In contrast, heating seemed to be independent of the material the trapped particle was made of. Figure 2e shows the results for microspheres of similar sizes (2.19 μm, 2.32 μm and 1.87 μm) but different dielectric materials (melamine resin, silica and polystyrene, respectively). Despite the ratio *α*/*K* for the three beads differing by 4 orders of magnitude (13.8 °C/W, 3.6·10^−3^ °C/W, 50 °C/W, respectively), *B* was found to change by only 0.1 °C/100 mW (±4%), in accordance with the fact that trap heating is mainly governed by laser absorption in the surrounding buffer.

### Simulations: heat transport

Temperature, *T*, is in general terms governed by the heat equation:

3 $${\nabla }^{2}T=\frac{1}{k}\frac{\partial T}{\partial t}-\frac{q}{K}$$

where *k* = *K*/*c*·*d* is the thermal diffusivity, *c* is the specific heat, *d* is the density, *K* is the thermal conductivity and *q* the energy absorbed per unit of volume and unit of time. Eq. 3 describes how energy transferred by the laser is diffused throughout the surrounding space.

To solve this equation, we need to specify both the function *q* and the boundary conditions. We used the absorption proposed by Peterman *et al*., which is an improved version of the spherical model of Liu *et al*.^2^, taking into account the finite volume of the focus^9^:

4 $$q\,=\,\frac{1}{2\pi }\frac{\alpha P}{{r}^{2}+{(\lambda /(2\pi N{A}^{2}))}^{2}}$$

Here, we used *f*(*θ*) = 1/2π (see section “Theoretical model” in ref.^9^) and we included the explicit dependence on the NA. In the equation, *α* (m^−1^) corresponds to the optical absorption and *P* to the incident laser power. Considering the geometry of the problem, defined by the flat parallel coverslips, with higher conductivity (1.4 W/m/K) and lower absorption (0.005 m^−1^) than water, acting as heat sinks, we alternatively chose boundary conditions with cylindrical coordinates, in which the spherical radial coordinate in Eq. 4 is expressed as ${r}^{2}={\rho }^{2}+{(z-{z}_{{\rm{trap}}})}^{2}$. We set the Dirichlet boundary conditions: Δ*T* = 0 at *ρ* = 80 μm and *z* = 80 μm; whereas a real water-glass interface was simulated at *z* = 0, which is the surface closest to the optical trap in all the experiments. The trap height, *z*_trap_, was 10 μm in all the experiments, unless otherwise stated.

**Figure 3:**
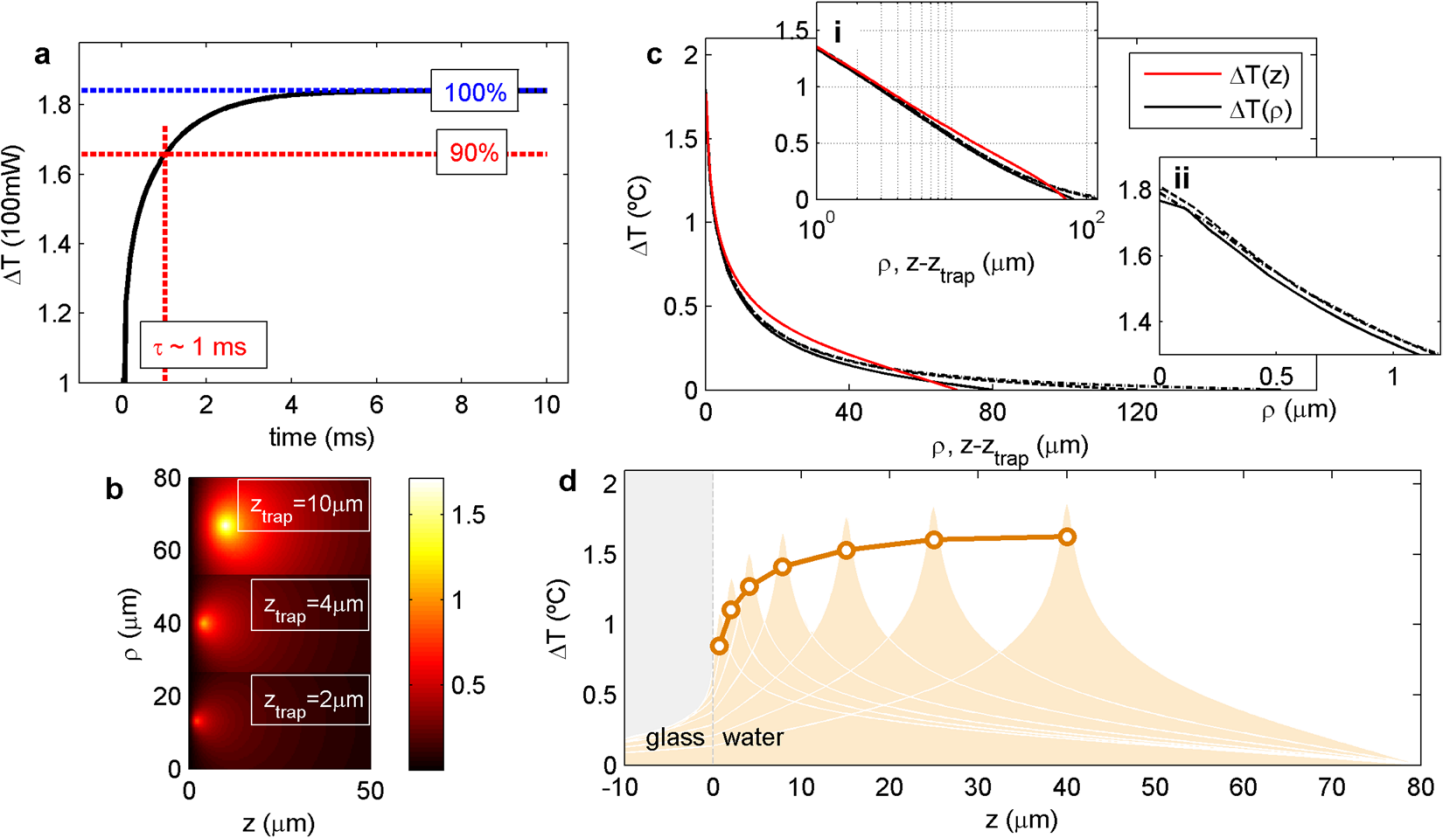
Simulation of temperature distribution inside the microchamber. **(a)** Temporal evolution of heating. The simulation shows that the temperature reaches 90% of its steady-state value after 1 ms. **(b)** Temperature distribution for three different axial positions of the trap. *z* = 0 represents the bottom surface of the chamber and *ρ* has been shifted appropriately for visualization. **(c)** Radial (black) and axial (red) temperature distributions following the ln(1/r) decay (log scale plot in inset i). Radial temperature distributions for the Δ*T* = 0 boundary condition at *ρ* = 80, 120 and 160 μm (solid, dashed and dot-dashed black lines) converge to the same value at short distances (inset ii). **(d)** Simulations of the temperature distribution at different heights (shadowed areas). The circles correspond to estimations of *B* for a 1.16-μm bead according to *B*_*bead*_ = Δ*T*(*ρ* = *R*_*bead*_).

The evaluation of the temporal part of the equation shows that after 1 ms the temperature reaches 90% of the steady-state value (Fig. 3a). For the highest flow velocity applied in our experiments (320 μm/s for 0.61-μm beads in water; see Supplementary Table 1), such a characteristic heating time is still faster than the fluid displacement time around the focal region. Sample heating of more than 90% is thereby ensured and a steady-state situation can be considered due to our precision in temperature measurements, which was around 10–15%. This agrees with the results in Supplementary Figure 4, which show no substantial variation in the measured viscosity for flow velocities up to 700 μm/s. For the lower flow rates applied to larger microbeads, the rate of heat diffusion is even faster compared with the velocity of the medium.

In Fig. 3b, we represent a typical *ρ* – *z* section of the temperature distribution around the trap (NA = 1.2) for three different axial positions of the focus. This shows that sample warming decreases as the trap approaches the water-glass interface, due to heat dissipation into the coverslip becoming more efficient. The axial and radial profiles for the trap at *z*_trap_ = 10 *μ*m shown in Fig. 3c reproduce the characteristic ln(1/*r*) decay proposed by Mao *et al*.^12^, whose solution had been empirically found by Celliers *et al*.^8^ some years before, Δ*T*(*r*) = *a* – *b*ln(*r*), and experimentally proved by Haro-González *et al*.^4^.

We can observe how the distance to the closest glass surface governs the heating produced by the laser (Fig. 3d). This is due to the low *α*/*K* of glass, which dissipates the heat produced by the laser, keeping the water-glass interface almost at room temperature. Unlike the result found by Peterman *et al*. obtained assuming spherical symmetry (see Supplementary Figure 7 and Supplementary Table 3), temperature was found to increase only over the first 0–10 *μ*m from the bottom surface of the chamber, where the heat dissipated by the glass coverslip was significant enough to cool down the focus. After this, *B* becomes almost constant as the distance increases across the rest of the chamber. As mentioned above, the temperature profile decaying sufficiently beyond 10 μm from the trap centre (Fig. 3c) leads to the cooling by heat dissipation through the coverslip being unnoticeable for *z*_trap_ values of more than 10 μm.

**Figure 4:**
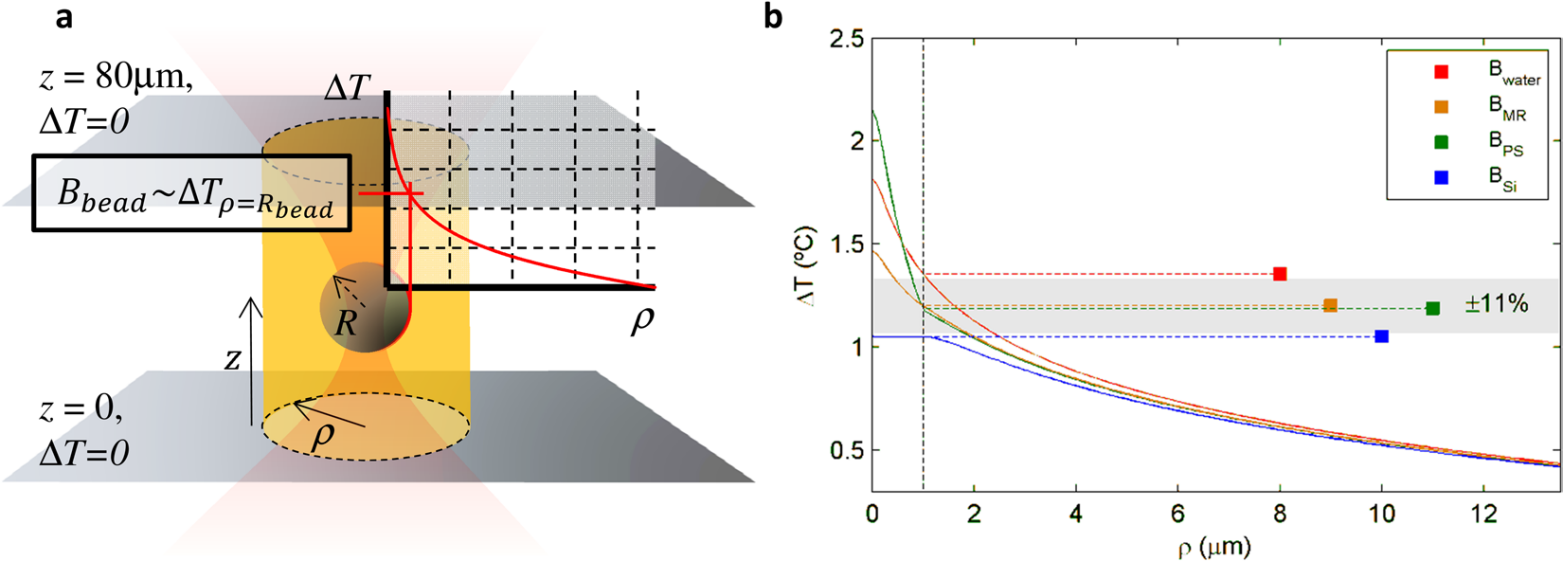
Simulation of temperature distribution inside the microchamber. **(a)** Relation between the heating rate for a given bead, *B*_bead_, and the temperature distribution produced by the trap. **(b)** Simulated temperature profile introducing a 2-μm microsphere at the focus of the trap. The grey area corresponds to the reproducibility of our measurements, shown in Fig. 2a. (MR: melamine resin, PS: polystyrene, Si: silica).

In contrast, the boundary in the radial coordinate, *ρ*, seems to have little impact on the value of the temperature near the focus. In Fig. 3c, inset ii, we show three temperature distributions simulated with the Dirichlet Δ*T* = 0 °C condition fixed at different distances, which collapse into a single curve, i.e., maintaining the same maximum heating in the vicinity of the trap. Interestingly, this profile describes the temperature increase measured for microspheres of different radii, *R*_bead_, as shown in Fig. 2c. We found a certain connection between the temperature at distance *R*_bead_ and the heating of a particle with that radius, i.e. between *B*_bead_ and Δ*T*(*ρ* = *R*_bead_) at *P = *100 mW (Fig. 4a). This is consistent with the fact that our method, based on drag force measurements, detects viscosity changes at the interface between the bead and the medium.

Concerning the effect of the NA, we had observed (Fig. 2c) considerably higher heating rates for the oil-immersion, NA = 1.3 objective, particularly on the smallest, 0.61-μm beads. Likewise, Fig. 2d showed large variations in *B* when modifying NA_eff_. The heat source in our simulations, *q*, includes the dependence on the NA and also yields greater heating for higher NA; though the resulting difference is one order of magnitude smaller than in the experiments. We believe that the model does not accurately describe the temperature increase at distances comparable to the beam waist, *r* ~ *w*_0_, as the result depends on the shape of the beam, which is not correctly modelled in this region. Additionally, experimental modification of the effective NA was achieved by reducing the beam diameter with an iris placed at an optical equivalent of the entrance pupil of the objective. This, at the same time, modifies the overfilling, which affects additional variables that govern the optical field at the focus and hence may also introduce some deviations from the simulations. For 3.00-μm beads, alternative model approaches^8,12^ qualitatively describe the heating–NA relationship (see Supplementary Figure 7). The fact that these beads are far larger than the beam waist, and that the optical field at *R*_bead_ is hence described more precisely, is the reason why the heating predicted through simulations is closer to the experimental results.

Finally, we checked the impact of the particle material on temperature. We incorporated into the simulations a second material with a spherical shape, corresponding to the particle, at the focus of the trap. We analysed the temperature distribution for three different dielectric materials (melamine resin, silica and polystyrene) and verified that the temperature over the particle surface, i.e. at a distance *R*_bead_, was almost independent of the material of which the microsphere was made within our experimental errors (Fig. 4b). Therefore, only the optical absorption and the thermal conductivity of the suspension medium seemed to play a role in the heating (see the ratio *α*/*K* for water and glycerol in Fig. 2c, inset). To a first approximation, an intuitive interpretation of these results is that the particle “experiences” on average the temperature of the surrounding solvent which is independent of the particle itself and is thereby given by the decreasing temperature profile produced by the trap alone, making the temperature increase lower for larger particles. Note that for absorbing particles, such as semiconductor or metallic particles, heating would be qualitatively higher since the energy transferred to the medium would be sufficient to alter the temperature distribution.

## Discussion

We demonstrated that the direct determination of viscosity changes caused by increasing laser powers is a robust strategy for obtaining local temperatures, i.e. the heating rate *B*. This was possible due to our force calibration being based on the detection of the trapping beam momentum (which is independent of the sample temperature and trapping power, among other features) which was compared to the temperature-dependent Stokes drag calibration. In this way, we could directly interpret changes in the measured force as variations in the sample temperature. Importantly, the method is precise enough to assess trap heating in watery buffers and makes it unnecessary to carry out experiments in media with higher *α*/*K* values.

The agreement between our results and simulations allows us to extract some interesting conclusions concerning the process of sample heating in an optical trap. The model proposed in ref.^9^ seems to capture the main elements necessary for the description of the behaviour of the temperature inside the chamber under different conditions. Simulations showed that the temperature distribution originated by an optical trap followed the typical ln(1/*r*) decay reported in other papers^4,8,12^. This decay describes the heating experienced by microspheres of increasing radii, which was observed to change by a factor of 2 and 3 from 0.61- to 3.00-μm beads, trapped with an NA = 1.2 and an NA = 1.3 objective, respectively. Such variation was considerably greater than the measurement precision, which was assessed to be of the order of 10–15%. In contrast, the material of the tweezed dielectric microspheres did not affect the measurement of *B*, which was also confirmed by simulations including the presence of the bead. Finally, the trap height played an important role in sample heating, as the coverslip acts as a sink, dissipating heat and maintaining the interface nearly at room temperature. When the trap was created below 10 μm, it was efficiently cooled down; whereas beyond that distance, *B* remained almost constant, due to the temperature increase being concentrated mainly within the 10 μm surrounding the trap focus.

The results of this work demonstrate the substantial variability in laser-induced trap heating, depending on the multiple experimental conditions examined. Temperature control is of the utmost importance in precise experiments in biophysics and other applications of optical trapping. This makes it highly necessary to perform *in situ* heating calibration, instead of applying the straightforward rule of thumb of 1 °C/100 mW. Large measurement inaccuracies, e.g. derived from erroneous thermal trap calibration, as well as changes in the activity of biological parameters^10^ could arise if the actual trap temperature is omitted. As a critical example, samples of a size similar to the beam waist, *R* ~ *ω*_0_, showed a ±2 °C/100 mW variation in *B* by only changing the effective NA of the trapping beam (Fig. 2d). Moreover, trap heating studied by other research groups with manifold techniques (see Supplementary Figure 6 and Supplementary Table 2) have demonstrated this variability. In conclusion, each experimental optical trap configuration leads to different heating performance due to several aspects, such as sample size, beam structure, microchamber dimensions and buffer specifications.

The remarkable heating observed under certain conditions could have an impact on experiments with cells. In such samples, laser radiation is absorbed by the intracellular medium, the cytosol, which is a complex and highly crowded compound. However, because cells and their components present weak absorbance in the NIR range, we can assume that absorption of laser radiation is dominated by water. Typical laser powers required to manipulate intracellular organelles are of the order of 200 mW. Natural structures inside cells are usually smaller than ~1 μm. In addition, high-NA (phase-contrast) oil-immersion objectives are normally preferred for the visualization of cells. Therefore, in general terms, heating will tend to be particularly large in this kind of experiments. Assuming that the heating of the cell is similar to that of water, and using the result for *B* for the smallest microsphere of diameter 0.61 μm and for NA = 1.3, we can estimate that the local temperature will rise by approximately 8 °C.

In fact, the large attainable temperature increase that 1064 nm lasers can produce could induce serious damage that one should assess beforehand. Although photodamage due, among other possibilities, to the generation of singlet oxygen is accepted to be the main source of damage upon live cells^19^, thermal heating should be reconsidered as a feasible origin of cell damage/death as well.

As we have shown, heating can be reduced by the use of trapping objectives of different NA or by creating the traps close to the coverslip. Furthermore, the use of laser wavelengths with lower optical absorption^20^, such as 820 nm, as proposed by Haro-González *et al*.^4^, appears to be a good choice for reducing photodamage and cell heating. In fact, those authors found that temperature increase at this wavelength was close to zero at 300 mW.

## Methods

### Optical trapping set-up and force measurements

The optical trapping set-up consisted of a CW Ytterbium fibre laser (IPG YLM-5-1064-LP, λ = 1064 nm TEM00, P_max_ = 5 W). The laser was directed into an inverted microscope (Nikon, Eclipse TE2000-E) through the epi-fluorescence port and focused on the sample plane by high-numerical-aperture objective lenses (Nikon CFI Plan Apo 60x water immersion, NA = 1.2 and Nikon CFI Plan Fluor 100x oil immersion, NA = 1.3). A telescope allowed us to adapt the beam waist to overfill the entrance pupil of the microscope objective. Overfilling (D_beam_/D_obj_) on the water- and oil-immersion objective was about 1 and 1.6, respectively. A dichroic mirror placed before the objective lens redirected the beam so the same path could simultaneously be used for the illumination light coming from the condenser, which passes through the mirror and reaches the CCD camera (QImaging, QICAM) at the bottom of the microscope.

The fibre laser was observed to oscillate in polarization, leading to possible power fluctuations when passing through polarizing elements, which were carefully avoided by properly setting the polarization of the beam (see Supplementary Figure 5).

Measurements of lateral trapping forces were carried out with a direct force-detection instrument (Impetux Optics, LUNAM T-40i), which collects the transmitted laser light from the sample with an NA = 1.4^15,16^ lens and tracks the light–momentum distribution at the BFP with a PSD. The 50-kHz PSD signals were processed with custom-designed analysis software (LabView and Matlab programs). As described in Results, lateral optical forces, *F*_X,Y_, were obtained through the PSD signals *S*_X,Y_ as *F*_X,Y_ = −*α*_detector_·*S*_X,Y_.

In turn, trap power was obtained as *P*_trap_ = 1/*ψ*·*S*_SUM_, where *S*_SUM_ is the SUM signal of the PSD and *ψ* (V/W) is the responsivity of the force-detection instrument. Briefly, it is calibrated by the manufacturer using an adaption of the dual-objective method from which the transmission of an objective can be obtained^16,21^.

### Sample preparation

All the experiments were conducted in a temperature controlled laboratory. The samples consisted of highly diluted solutions of polystyrene monodisperse spherical particles with diameters of 0.61 μm, 1.16 μm, 1.87 μm and 3.00 μm (refractive index *n* = 1.59, density *d = *1.05 g/cm^3^, all with a standard deviation of 2–3%), provided by the manufacturer and confirmed with dynamic light scattering for the smallest ones (see Supplementary Information). We additionally used melamine resin 2.19-μm beads and silica 2.32-μm beads for the analysis of laser heating on samples made of different materials.

As buffers, we used water (density, *d* = 0.99 g/cm^3^; absorption coefficient, α = 14.2 m^−1^; specific heat, *c = *4.18 J/g·K; and thermal conductivity, *K* = 0.6 W/m·K at 25 °C) or glycerol (density, *d = *1.261 g/cm^3^; absorption coefficient, α = 21.4 m^−1^; specific heat, *c = *2.41 J/g·K; and thermal conductivity *K* = 0.28 W/m·K at 25 °C). The experimental home-built microchamber consisted of a thick (1 mm) glass slide and a coverslip separated by 80 μm wide double-sided Scotch tape. Particles were trapped at *z*_trap_ = 10 μm from the lower coverslip-liquid interface, except for the experiment on axial dependence *B*(*z*), which was carried out with the water-immersion, NA = 1.2 objective to avoid spherical aberration due to the refractive index mismatch and ensure efficient trapping in depth. Surface interaction was compensated using Faxén’s coefficient^18^.

### Drag forces generated with a piezoelectric stage

We used a piezoelectric stage (Piezosystem Jena, TRITOR 102 SG) driven by a voltage sequence with controlled amplitude and frequency which produced, after calibration of the low-pass filtering of the electronics, triangular time signals with a precise slope. We set the flow velocity, *v*, to produce similar hydrodynamic forces on all the (different sized) beads we used: approximately 1.6 pN. Stokes’ law provides an analytical expression for the force applied on a microsphere (of radius *R*): *F*_drag_ = 6π*ηRbv*, where *η*(*T*) is the viscosity of the surrounding medium, which is a function of temperature, *T*, and *b* is Faxén’s coefficient to correct for surface hydrodynamic interaction^18^. The flow velocities were always below the rate of heat dissipation, to ensure stationary heating at the trap focus. See Supplementary Figures 2, 3 and 4 for a more detailed description.

### Temperature increase obtained from drag force measurements

Measurements of temperature increase due to laser heating were based on the determination of the light momentum changes when controlled forces were applied to trapped particles at different laser powers. The triangular flow oscillation produced by the piezoelectric stage yielded square force signals that were registered by the PSD of the force detection system. The constant force plateaus corresponded to the back-and-forth, ±6π*ηRbv* Stokes forces. The absolute drag force was calculated as the half-difference between the two averaged plateaus, which automatically cancelled out the initial momentum of the beam. To average the force values, approximately 10,000 data points were taken, corresponding to the timeframe over which the piezoelectric stage yielded a constant slope, i.e., constant drag force (see Supplementary Figures 2 and 3).

Each force value used for sample temperature calculations was taken as the mean over 20 consecutive cycles; and the corresponding standard deviation, typically in the range ±1–3%, was considered to be the associated error bar. Such standard deviation resulted in an uncertainty of ±0.5 °C–1.5 °C in temperature measurements. Ten temperature measurements, *T*_i_, at trapping powers, *P*_i_, of 20 mW, 40 mW, …, 200 mW, were linearly fitted by *T* = *T*_room_ + *BP*, from which we could obtain the heating rate, *B*, with an estimated precision of 10–15%. Final heating rates (Fig. 2) were calculated from the mean of 3–5 measurements.

### Trap heating simulations

To solve the temperature distribution due to an optical trap (see Eq. 4), we used an adaptation of the MathWorks Partial Differential Equation (PDE) Toolbox that allowed us to include the Jacobian for cylindrical coordinates (see Supplementary Information). Except for the study of temporal evolution, all simulations were carried out by solving the elliptic equation to obtain the stationary temperature distribution. For comparison with the experimental measurements of B (°C/100 mW) (Fig. 2), we obtained the temperature solutions, ΔT(*ρ*, z) at a laser power of 100 mW. See Results for a detailed discussion of the boundary conditions and the definition of the heat source *q*, as well as the main results of and conclusions derived from the simulations.

## Electronic supplementary material

Supplementary Information
