# Supplementary material for: Influence of experimental parameters on the laser heating of an optical trap
Source: Sci Rep. 2017 Nov 22;7:16052. doi: 10.1038/s41598-017-15904-6 (PMC5700206; doi:10.1038/s41598-017-15904-6)
Supplement: Supplementary file 1 — Supplementary Information [file 41598_2017_15904_MOESM1_ESM.pdf]

Supplementary material

Influence of experimental parameters on the laser heating of an optical trap

Frederic Català[1,2], Ferran Marsà[2,3], Mario Montes-Usategui[1,2,3], Arnau Farré[2,3], Estela Martín-Badosa[1,2*]

[1]*Optical Trapping Lab – Grup de Biofotònica, Departament de Física Aplicada, Universitat de Barcelona, Martí i Franquès 1, Barcelona 08028, Spain*
[2]*Institut de Nanociència i Nanotecnologia (IN2UB), Martí i Franquès 1, Barcelona 08028, Spain*
[3]*Impetux Optics S. L., Trias i Giró 15 1-5, Barcelona 08034, Spain*

**Corresponding author:* estela.martinb@ub.edu
http://biopt.ub.edu

1. **Relation between power spectrum and light momentum trap calibration with temperature**

2. **Generation of controlled, precise drag forces with a piezoelectric stage**

3. **Laser heating measurements in assorted optical tweezers laboratories**

4. **Heat transport simulations**

1. Relation between power spectrum and light momentum trap calibration with temperature

In previous work[16], we showed the validity of interpreting, under certain strict conditions, back focal plane (BFP) interferometry signals as measurements of light momentum changes; that is, as direct readings of the trapping force. The most significant requirements for such an interpretation are: 1) the use of a high-NA, aplanatic collecting lens that captures all the light from the optical traps; and 2) to track the light intensity distribution at the BFP of the collecting lens with a position sensitive detector (PSD). The usual approach to measuring forces in optical tweezers consists of calibrating the trap stiffness κ (pN/μm), in accordance with: $F = -\kappa{\cdot}x$, and the position sensitivity β (μm/V), such that: $x = \beta{\cdot}S_x$ (where S_x is the sensor positional voltage signal). In particular, we proved that if the aforementioned conditions hold, the product $\alpha_{\text{trap}} = \kappa \cdot\beta$ (pN/V) is invariant and equal to the constant and permanent momentum calibration of the sensor, $\alpha_{\text{detector}} = R_D/\psi f'c$, where R_D is the detector radius, f' and ψ (V/W) are the focal length and the responsivity of the instrument, respectively, and c the speed of light:

$$\alpha_{trap} \equiv \kappa \cdot \beta = \frac{R_D}{\psi f' c} \equiv \alpha_{detector} \qquad (1)$$

In contrast to α_{trap}, which must be calibrated, for example using the power spectrum method (Supplementary Ref. 1), α_{detector} is obtained from first principles and is determined by the optical parameters of the beam detection system alone. This suggests that, if the required instrument design conditions are fulfilled, no new *in situ* trap calibration is necessary when the experiment changes[15,16] and force can be directly obtained as: $F_{x,y} = -\alpha_{\text{detector}}{\cdot}S_{x,y}$. The momentum calibration of the force sensor, α_{detector}, is independent of the geometry of the trapped object and of the structure of the trapping beam[17]; moreover, and importantly for the purpose of this paper, it is not dependent on laser power or chamber temperature.

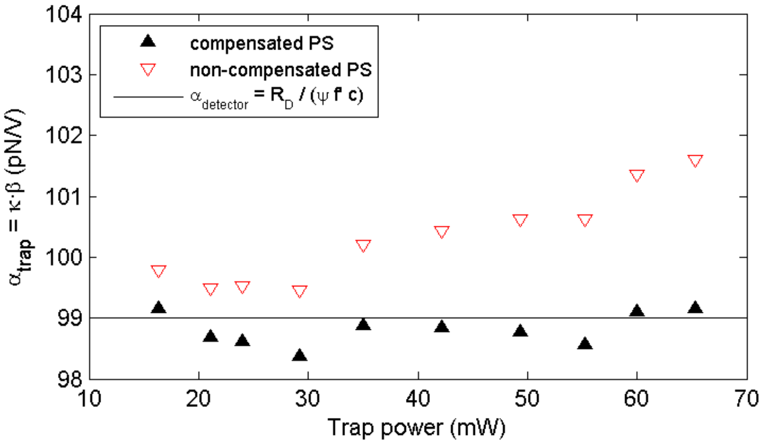

Figure S1 | Discrepancies between α_{detector} and $\alpha_{\text{trap}}=\kappa{\cdot}\beta$, obtained from the power spectrum analysis, provide evidence of change in the sample when the laser power is increased. When the temperature increase is considered in the power spectrum (PS) fitting (Supplementary ref. 1), the calibration is compensated and α_{trap} is constant.

In Fig. S1, we used the discrepancy between the two schemes to determine changes in sample temperature. A close look at the results for $\alpha_{\text{trap}} = \kappa \cdot\beta$ reveals that the equivalence $\alpha_{\text{trap}} = \alpha_{\text{detector}}$ starts to fail when the laser power is increased, with α_{trap} deviating from the constant value $R_D/\psi f'$c. This is indicative of a local temperature increase due to laser absorption (of 4 ºC/100 mW in Fig. S1), which leads to incorrect κ and β calibration if overlooked. As discussed by Peterman *et al.*[9], temperature affects the power spectra of optically trapped microspheres, both as a thermal variable governing Brownian motion and through the viscosity of the solvent, which importantly is dependent on it.

2. Generation of controlled, precise drag forces with a piezoelectric stage

The piezoelectric stage was moved with constant velocity, $v = dx_{piezo}/dt$, producing steps at $-6\pi\eta Rbv$ and $+6\pi\eta Rbv$ of constant drag force (Fig. S2). The mean force was obtained by averaging the signal (with $f_{sampling}$ = 50 kHz) over $n \sim$ 20 steps, taking only ~10,000 points from each step, which corresponds, as we will discuss below, to the range where the velocity was indeed constant. For an accurate calculation of local viscosity from Eq. 1 (see Main Text), we need to know the velocity of the piezoelectric stage with high precision.

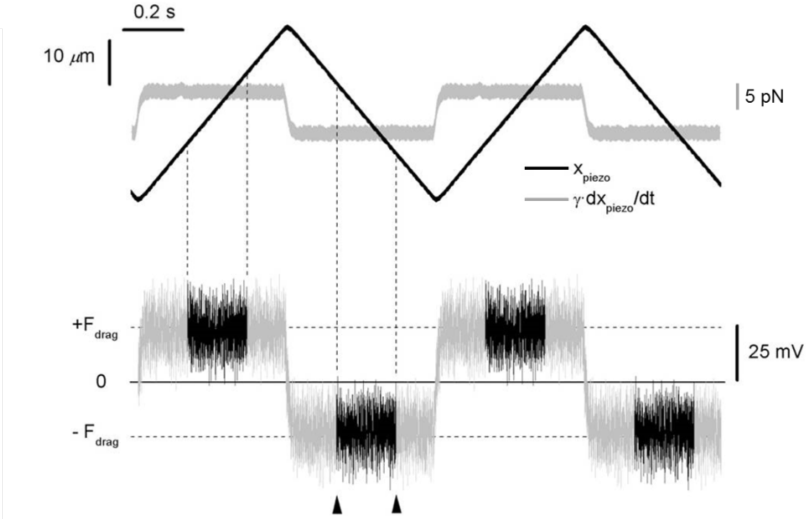

Figure S2 | Constant force on trapped samples. Top – Typical triangular trajectory drawn by the piezo-stage (black) and it's derivative (grey), i.e. the velocity of the surrounding medium. Bottom – force signal from a trapped sample, which is proportional to the velocity of the piezo-stage. In black, the point series considered for averaging the force measurement.

We studied the transfer function of the control electronics and found that, even at low frequencies (1-10 Hz), the amplitude of the output voltage sent to the stage differed by ~10%-20% from the input signal (see Fig. S3a). The ratio of the two values was quite independent of the amplitude, but decreased with frequency following a single-pole-like function with a roll-off frequency of 20 Hz. The stage velocity therefore had to be corrected to take into account the deformation introduced in the triangular signals by the low-pass filtering of the electronics (see Fig. S3b).

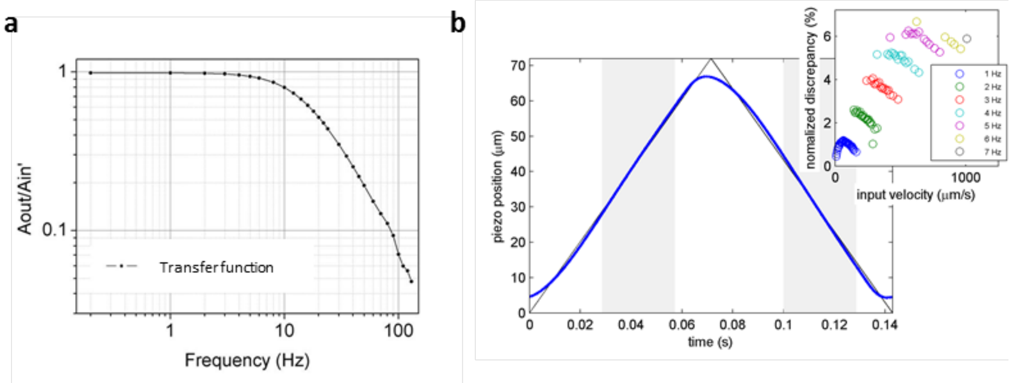

Figure S3 | (a) Amplitude transfer function of the piezoelectric platform. **(b)** Output signal monitored through an NI-DAQ interface (blue line) showing certain deviation from the desired oscillation (black line). In the inset: deviation of the actual constant velocities (obtained from linear fits over the grey areas in **b**), for oscillation frequencies from 1 to 7 Hz. Every colour corresponds to a different oscillating frequency.

To do this, we correlated the monitored output voltage with the triangular input signal and we found that only in a region of 40%-80% of the semi-period (shadowed area) was the velocity of the stage constant. Moreover, the

actual velocity was larger than the theoretical value and it depended on the amplitude, *A*, and frequency, *f*, of the signal (see Fig. S3b (inset) and Table S1). All measurements were kept below 6 - 7 Hz to ensure a constant velocity time frame. In Eq. 1 (see Main Text), we used this calibrated velocity value for the calculation of the viscosity change.

Furthermore, we analysed the variability of the measured viscosity with the stage velocity and found no significant change, which indicated that the dissipation of heat was faster than the motion of the fluid, so the temperature "experienced" by the particle was constant (Fig. S4).

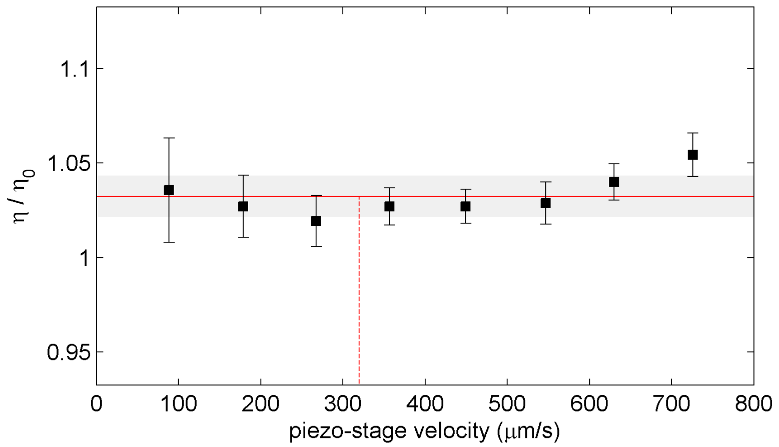

Figure S4 | Water viscosity measured with a 0.61-μm bead at different flow velocities. The vertical dashed red line indicates the velocity of 320 μm/s applied for this kind of beads in our study (Table S1). The solid red line and the grey shadow are the mean and standard deviation of the quotient η/η_0.

The oscillation parameters were chosen so that they produced similar drag forces on the microspheres used, which had different radii and were given by their corresponding manufacturers (Table S1). The diameter of the smallest microspheres (0.61 μm) was also confirmed using dynamic light scattering (DLS).

Bead (μm)	Velocity in water				Force at 25ºC (pN)	Velocity in glycerol (μm/s)	Force at 25ºC (pN)
	A (μm)	f (Hz)	target (μm/s)	actual (μm/s)			
0.610 ± 0.014 (PS)	40	2	320	323.1	1.68	0.4	2.14
1.16 ± 0.04 (PS)	40	1	160	160.6	1.62	0.2	2.05
1.87 (PS)	25	1	100	100.8	1.67	-	-
2.19 ± 0.05 (MR)	21	1	84	84.7	1.66	0.16	3.15
2.32 (Si)	20	1	80	80.7	1.68	-	-
3.00 ± 0.07 (PS)	16	1	64	64.6	1.78	0.12	3.27

Table S1 | Actual flow velocities applied to the different microspheres used in our drag experiments, measured via the piezoelectric stage output reading, and theoretical Stokes drag forces applied.

Finally, we checked the long-term stability of our fibre laser output power. We observed large oscillations (10%-20%) for long periods of time (see Fig. S5), whose origin was the fluctuation in polarization of the beam. These oscillations vanished when no polarizing elements were introduced along the optical path, or by orientating the beam polarization parallel to the transmission axis of the polarizing beam splitter.

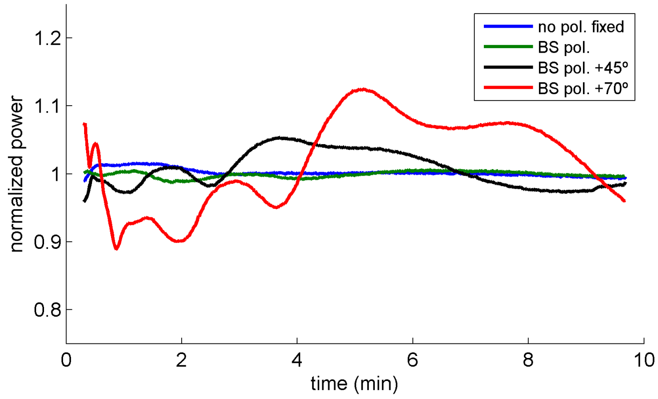

Figure S5 | Laser power fluctuations under different polarization states: free of polarizing elements (blue), linearly polarized parallel to the beam splitter (BS) transmitting direction (green), and at 45º (black) and 70º (red) with respect to that direction.

3. Laser heating measurements in assorted optical tweezers laboratories

A large number of studies have been carried out to assess the phenomenon of sample warming due to infrared laser absorption in optical tweezers. In Fig. S6 and Table S2, we indicate some of the reported results that represent the state of the art in this matter. It is of special significance that the strategies undertaken are based on a wide variety of principles.

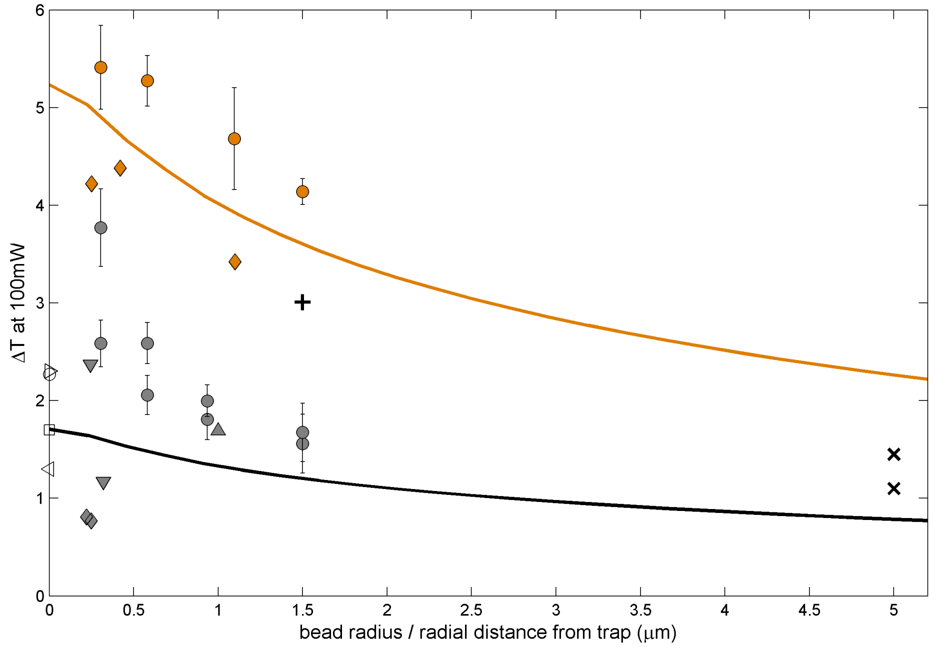

Figure S6 | Compilation of sample heating measurements in the literature obtained via a number of different methods. The black (orange) line corresponds to the FDTD simulations in water (glycerol) discussed in Results (see Main Text).

First, several heating studies have been based on the temperature dependence of the buffer viscosity (either water or glycerol), which can be determined with a microsphere trapped in the optical trap through direct drag force measurements based on the trapping light momentum[12], power spectrum calibration[9] or active-passive calibration[11]. Second, one can find studies consisting of sample thermometry through thermally-dependent fluorescence

performed on different optically-trapped samples[2,3,4]. Thirdly, experiments on empty traps based on different approaches have also been undertaken[5,6,7,8].

**Author**		**Technique**	**λ_{trap} (nm)**	**Target**	***B* measurements (ºC/100 mW)**			**B value at *λ=1064 nm***
					**H_2O**		**Glyc**	
					NA1.2	NA1.3	NA1.2	
Català	●<br>●	Light momentum & Stokes drag	1064	0.61 μm PS<br>1.16 μm PS<br>1.87 μm PS<br>2.19 μm MR<br>2.32 μm Si<br>3.00 μm PS	**2.59**<br>**2.06**<br>**1.81**<br>1.93<br>1.90<br>**1.56**	**3.77**<br>**2.59**<br>**2.00**<br>--<br>--<br>**1.67**	**5.41**<br>**5.27**<br>--<br>**4.68**<br>--<br>**4.14**	--
Peterman[9]	◆<br>◆	Power spectrum & Stokes drag	1064	0.444 μm Si<br>0.500 μm Si<br>0.502 μm PS<br>0.840 μm PS<br>2.200 μm PS	**0.81**<br>**0.77**<br>--<br>--<br>--		--<br>--<br>**4.22**<br>**4.38**<br>**3.42**	--
Mao[12]	▲	Light momentum & Stokes drag	975	2 μm Si	5.6		--	**1.69**
Jun[11]	▼	Active-Passive	980	490 nm PS<br>642 nm Si	7.8<br>3.8		--	**2.37**<br>**1.17**
Haro-González[4]	+	Quantum dot luminescence thermometry	750<br>808<br>920<br>980<br>1090	3 μm PS	0<br>0<br>5<br>9.9<br>4.9		--	0<br>0<br>8.26<br>**3.01**<br>4.30
Liu[2,3]	×	Temperature-dependent luminescence (Laurdan)	1064	sperm cells<br>CHO cells (10 μm)<br>liposomes (10 μm)	1<br>**1.15**<br>**1.45**		--	--
Celliers[8]	○	Refractive index	985	--	4 K/55 mW		--	**2.27**
Kuo[7]	□	Wax melting	1064	--	**1.7**		--	--
Ebert[5]	◁	Temperature-dependent luminescence (Rhodamine B & 110)	1064	--	**1.3**		--	--
Wetzel[6]	▷	Temperature-dependent luminescence (Rhodamine B & 110)	1064	--	**2.3**		--	--

Table S2 | Assorted studies of laser heating in optical traps. From left to right, we indicate: the first author of the publications reporting the results, the symbol used in Fig. S6, the method used to assess trap temperature, the laser wavelength used, the sample trapped (size and material), the values reported (subsections NA = 1.2 and NA = 1.3 for the present study, and measurements in glycerol buffer as well for measurements by Peterman *et al.*). In the last column, we indicate the equivalent *B* factor obtained by applying the Beer-Lambert law[13] with spectral attenuation $\alpha(\lambda)$ reported by Kedenburg *et al*[20]. Values represented in Fig. S6 are in bold typography. PS: polystyrene, MR: melamine resin, Si: silica, CHO: Chinese hamster ovary.

4. Heat transport simulations

We made use of the MathWorks Partial Differential Equation (PDE) Toolbox to simulate the heating of the sample due to a laser trap. Given that the geometry we used exhibits cylindrical symmetry, we adapted the heat equation to include the Jacobian along the radial component and solved the problem on a 2D surface.

As mentioned in the Main Text, the modelling adopted by Peterman *et al.* of $B(z)$[9], which was conceived in spherical geometry in which the trap is created at $r = 0$ and the $\Delta T = 0$ condition is fixed at $r = z$, exhibits a non-stopping increase (Fig. S7a). In contrast, the choice of cylindrical symmetry and the Dirichlet boundary conditions at two parallel surfaces corresponding to the coverslips yields a constant B value after an abrupt rise over the first 10 μm (Fig. S7b). This is especially evident in the real sink case with thermal conductivity K_{glass} (Fig. S7c), due to the coverslip only being capable of cooling the sample sufficiently when the trap is placed very close to the interface.

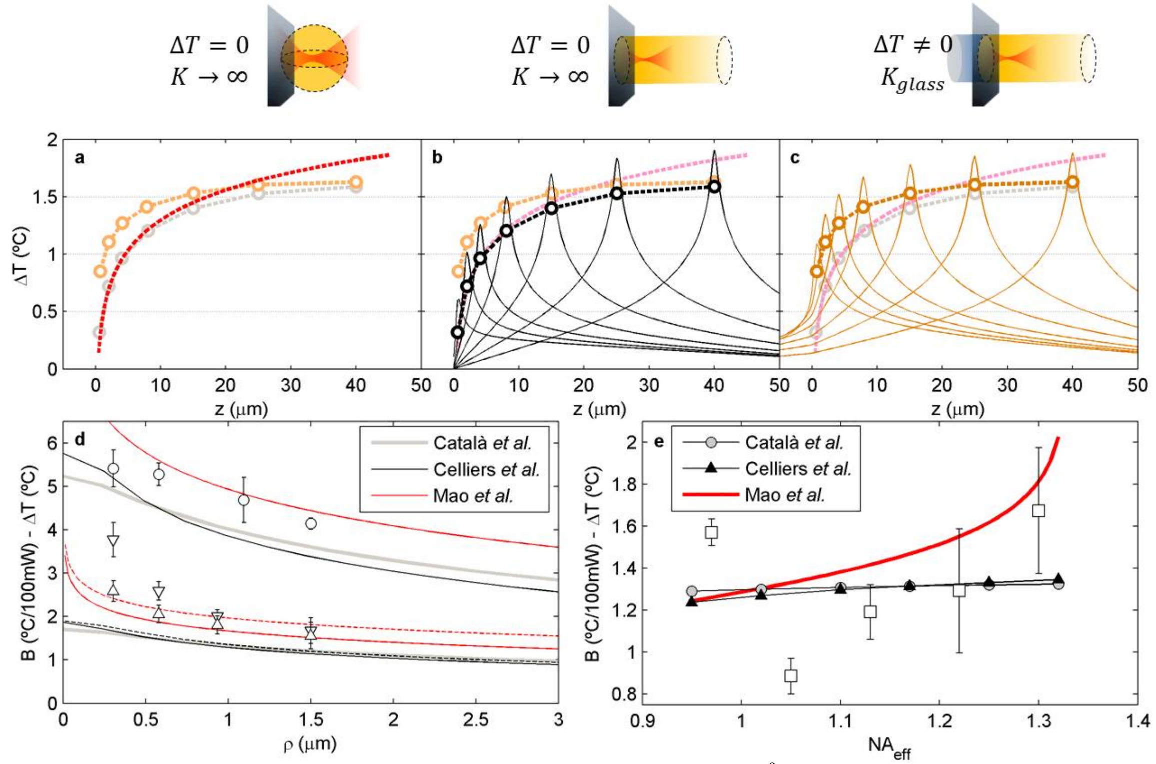

Figure S7 | Heat transport simulations. (a) The model of Peterman *et al.*[9] of $B(z)$ simulated in spherical coordinates (red dashed line). **(b)** $\Delta T(z)$ distributions in cylindrical geometry with $\Delta T = 0$ at $z = 0$ and 80 μm (solid lines correspond to $\Delta T(z)$ curves and circles are estimations of B for a 1.16-μm microbead). **(c)** Real sink at $z = 0$ and $\Delta T = 0$ at $z = 80$ μm. In **a-c**, the other two curves are superimposed in a light colour for comparison. **(d)** Models in refs. 9 (grey), 8 (black) and 12 (red) simulated in our cylindrical geometry. Solid (dashed) lines are simulations with NA = 1.2 (NA = 1.3). **(e)** Simulation of the dependence of B on the NA for the three models as in **d**, for 3.00-μm microbeads.

In Fig. S7d and Table S3, we show two models in the literature that describe the radial temperature profile with similar accuracy. The model of Celliers *et al.*[8] was simulated in the same cylindrical geometry and coincided closely with our simulations, with a slightly greater temperature increase, $\Delta T(\rho)$, for the NA = 1.3 objective than for the NA = 1.2 objective. The analytical expression provided by Mao *et al.*[12] also coincides with our measurements and exhibits an even greater difference between the two objectives.

Català	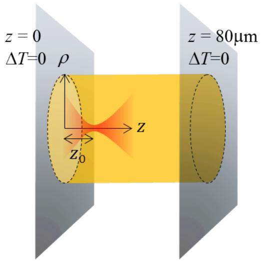	$q = \frac{\alpha P}{2\pi} \frac{1}{\rho^2 + (z - z_{trap})^2 + a^2}$
Peterman[9]	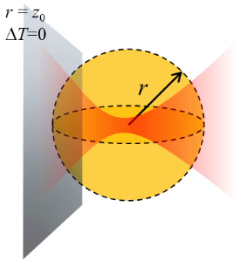	$q_{Peterman} = \frac{\alpha P}{2\pi} \frac{1}{r^2 + a^2}$
Celliers[8]	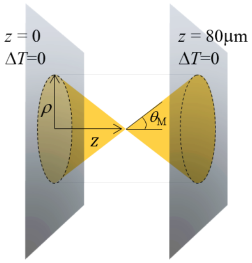	$q_{Celliers} = \begin{cases} \frac{\alpha P}{2\pi(1-\cos\theta_M)} \frac{1}{\rho^2 + z^2}; & \frac{\rho}{z} < \tan^{-1}\theta_M \\ 0 & ; \frac{\rho}{z} > \tan^{-1}\theta_M \end{cases}$
Mao[12]	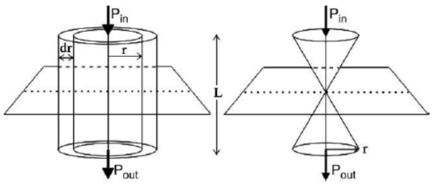 *Figure in ref. 12.*	$\Delta T(\rho) = \frac{\alpha P}{2\pi K} \ln \frac{R}{\rho}$

Table S3 | Laser heating models and geometries. Different geometries and mathematical models have been used for laser heating simulations.

Finally, we simulated the effect of reducing the effective NA of the trap (Fig. 2d and Fig. S7e). Although expressed in terms of the trap power, the heating rate, B (ºC/100 mW), is more related to the local irradiance, which eventually explains the observed variation. Irradiance is contained in the shape of the heating source, $q(r)$, in models of Peterman *et al.*[9] and Celliers *et al.*[8], as well in the R factor in that of Mao *et al*[12].

Models in refs. 8 and 12 seem to capture the main behaviour of the heating dependence on NA_{eff} for the 3.00-μm beads. As compared with the experimental measurements, they reveal an ascending pattern that can be directly connected to the wider light cone illuminated, i.e. wider heat source. For the smaller, 0.61-μm beads, one could think of the smaller beam waist created with higher NA_{eff} to conclude that the therefore higher irradiance leads to greater heating as well. However, as mentioned in Results (see Main Text), the optical field at the bead-medium interface is here bound to a number of aberrations and deviates from an ideal Gaussian shape, which leads to the measurements notably deviating from the simulations. Besides, our reducing NA_{eff} by means of a diaphragm at the back of the trapping objective leads to a different overfilling, thereby producing higher variations in the local optical field.

Supplementary References

1. Berg-Sørensen K. & Flyvbjerg H. Power spectrum analysis for optical tweezers. *Rev. Sci. Instrum.* **75**, 594–612 (2004). (doi: 10.1063/1.1645654)
